# Solar Panels String Predictive and Parametric Fault Diagnosis Using Low-Cost Sensors

**DOI:** 10.3390/s22010332

**Published:** 2022-01-03

**Authors:** Emilio García, Neisser Ponluisa, Eduardo Quiles, Ranko Zotovic-Stanisic, Santiago C. Gutiérrez

**Affiliations:** 1 Instituto de Automática e Informática Industrial, Universitat Politècnica de València, Camino de Vera, s/n, 46022 Valencia, Spain; egarciam@isa.upv.es (E.G.); neipon@posgrado.upv.es (N.P.); rzotovic@isa.upv.es (R.Z.-S.); 2 Instituto de Diseño y Fabricación (IDF), Universitat Politècnica de València, Camino de Vera, s/n, 46022 Valencia, Spain; scgutier@mcm.upv.es

**Keywords:** solar panel, predictive maintenance, fault diagnosis, photocell, partial shading degradation, ESP8266, SCADA, iFIX

## Abstract

This work proposes a method for real-time supervision and predictive fault diagnosis applicable to solar panel strings in real-world installations. It is focused on the detection and parametric isolation of fault symptoms through the analysis of the Voc-Isc curves. The method performs early, systematic, online, automatic, permanent predictive supervision, and diagnosis of a high sampling frequency. It is based on the supervision of predictive electrical parameters easily accessible by the design of its architecture, whose detection and isolation precedes with an adequate margin of maneuver, to be able to alert and stop by means of automatic disconnection the degradation phenomenon and its cumulative effect causing the development of a future irrecoverable failure. Its architecture design is scalable and integrable in conventional photovoltaic installations. It emphasizes the use of low-cost technology such as the ESP8266 module, ASC712-5A, and FZ0430 sensors and relay modules. The method is based on data acquisition with the ESP8266 module, which is sent over the internet to the computer where a SCADA system (iFIX V6.5) is installed, using the Modbus TCP/IP and OPC communication protocols. Detection thresholds are initially obtained experimentally by applying inductive shading methods on specific solar panels.

## 1. Introduction

The European Union (EU) has proposed a more exhaustive analysis of the use of photovoltaic solar energy in its future energy policy. Although in recent times the most significant increase in the demand for solar energy was taking place in Asian countries, 2019 has been the year that has marked a difference, in which in the EU this demand has experienced higher growth, recovering a leadership position compared to other solar regions, by installing more solar energy than that based on other renewable energy technologies.

Currently, the main reason for the success of solar energy in the EU is its low cost. Solar energy is often cheaper than any other current technology. This is true for retail electricity and, increasingly, for wholesale power as well. With the steeper cost reduction curve ahead, the competitiveness of solar PV will increase even more. Other international markets outside the EU also have the opportunity to benefit from the cost advantage of solar energy.

Solar energy has achieved comparatively lower costs than its competing renewables, and this downward trend will be more pronounced in the near future, guaranteeing very promising success. Additionally, Brussels, with its 2019 “Clean energy for all Europeans’’ legislative package, favours the diffusion of photovoltaic energy until 2030, given its versatility, speed and ease of installation.

The most profitable way to decarbonise the EU economy in the short term, contributing to the achievement of its energy objectives and avoiding climate change, is to undertake electrification based on renewable energies, where solar energy is in a very prominent position. Due to its versatility, capacity, and the reduction in prices of solar panels of 96% in the last ten years, solar energy is suitable and profitable to supply direct renewable energy to public buildings, homes, companies, businesses, farms, agricultural facilities, charging stations, etc.

Also, gases such as hydrogen obtained from renewable energies should contribute to the fulfilment of the European Green Agreement in 2050, especially to be used in sectors that are difficult to decarbonise, such as energy-intensive industries and heavy transport. To produce renewable hydrogen, the sector requires a political commitment and regulatory framework to end new investments in conventional fossil fuel-based generation and redirect all available financing to generate a genuinely renewable hydrogen economy [1].

With the aim of continuing to increase the competitiveness of solar energy, there are possible additional actions to be taken in relation to improving the operating and maintenance costs of solar installations, based on the application of more appropriate advanced methods for the supervision and predictive fault diagnosis of the facilities. It must be taken into account that aging due to superimposed causes of degradation, as happens with other types of industrial processes in direct outdoor installation conditions, is a natural and inexorable phenomenon that becomes a major problem for any photovoltaic installation. These installations are typically exposed to inclement weather conditions (such as solar radiation, cold, rain, dust, humidity, snow, wind, and electrical storms) or pollution. Such conditions make the appearance of the degradation phenomenon unavoidable, which can significantly decrease energy production, diminishing the economic benefits expected from the installation, increasing the expected return time of the investment and introducing a component of uncertainty in the establishment of the warranty period. All of these are fundamental factors that make it difficult for photovoltaic technology to continue increasing its competition with other types of energy.

In the opinion of the authors of the present work, the conventional monitoring and diagnosis methods that have been used in photovoltaic installations have not been the most suitable since they were applied focused on the inverters of the panel strings [2,3,4,5] but not on the solar panels themselves individually. However, it is in the panels where the effects of degradation and catastrophic failures mostly take place in an indisputable way, not making possible the objectives of the immediate detection and isolation of the degrading event. This problem in all types of photovoltaic installations can be decisive to know on which panel to act immediately, to disconnect the affected panel, improve performance, or avoid the event of catastrophic failure. At present, the reasons that were argued for the high costs necessary to carry out monitoring focused on the solar panel, taking into account the technological development experienced in low-cost sensors and devices, cannot be justified [6,7].

In this work, a predictive fault diagnosis method has been applied to be used in the general prevention of the degradation of the panels in photovoltaic installations. It is designed, in a special way but not exclusively, for the predictive diagnosis of the phenomenon of the appearance of hot spots, which is recognized as one of the main causes of solar panels degradation.

For the implementation of the predictive fault diagnosis algorithm, a specific low-cost architecture has been developed to carry out online supervision of the predictive fault parameters in each of the solar panels of the solar installation, which allows the automatic disconnection of the panel affected avoiding the accumulation of the degrading effect. This architecture is integrable and scalable with conventional distributed control systems of medium and large photovoltaic installations, which will allow achieving better operating and maintenance costs.

In the method, for the best estimate of the quantitative threshold value predictor of the degrading symptom, the performance of experimental inductive shading tests is included to correlate the reduction in power achieved with the induced shading. With this, an adequate threshold value of the power deviation can be estimated so that it can be used as a predictor parameter, avoiding the reaching of phases of irrecoverable deterioration of the solar panel.

The main goal of the contribution is to develop a diagnosis method for PVM that is predictive, based on the online detection of a predictor symptom, centred and sampled on each solar panel of the string, instead of being centred on the inverter, low-cost implementable, and integrable in actually working photovoltaic installations.

This document is distributed as follows: Section 2 reviews works referring to the most common failures in PV panels and works similar to the one proposed. In Section 3, the hardware and software materials and the methodology to carry out the work are described. In Section 4, the results obtained are shown, and a discussion of the results obtained with this proposed methodology is carried out. Finally, in Section 5, the conclusions and possible improvements that could be made are indicated.

## 2. Review of Solar Panels Fault Diagnosis Methods

Solar panels, when installed outdoors, are exposed to different degradation phenomena due to environmental conditions (rain, hail, snow, cloudy days), vandalism, dust, bird droppings, shadows from buildings near the facilities, aging, etc. Each of these types of degradation phenomena can produce faults and failures [8,9,10], causing severe and irreparable damage such as cracks, hot spots, fires, and total loss of the solar panel. For this reason, it is necessary to carry out supervision and maintenance to ensure reliability, efficiency and extended useful life in order to obtain maximum performance in power generation [11,12].

Degradation failures due to environmental factors is the biggest problem faced by solar panels; for this reason, several studies have been carried out on solar panels fault diagnosis.

From the point of view of the application of an early, systematic, online, automatic, permanent predictive diagnosis, and supervision method, some requirements are desirable, such as (a) high sampling frequency as fast as desired, (b) it should be based on the supervision of an accessible predictive electrical parameter, (c) the detection occurs with an adequate margin of manoeuvre so that it is able to alert and stop the degradation phenomenon that causes the development of a future failure, and (d) it can be used to place the system in a state of alert against the appearance of a diverse set of characteristic faults, activating the detection, isolation, and analysis phases recognized in the general concept of fault diagnosis [7].

Although previously the I–V parameters have not been taken into account for the application of the supervision and diagnosis located in each of PVMs, justified by the argument of the increase associated with the costs of the investment to be made, currently with the experienced development of low-cost IoT sensors, this argument of increased costs is not justified. The reduction in the power detected in the PVIs, especially the one focused at the level of each of the panels, meets the best conditions to be used in the application of predictive diagnosis techniques. Specifically, those parameters involved in the reduction of the power generated in each of the PVMs, such as I-V, are strong candidates to be supervised online in the form of time series in order to apply trend analysis algorithms on the comparative deviation to obtain indicative signs prior to reaching a failure condition without recovery possibility. Traditionally, the detection of the decrease in power in PVIs has been carried out in the inverter connected to each of the strings of PVMs, but although the accumulated decrease in the power produced can be detected in the inverter, the possibility of quickly achieving the immediate objective of the detection and fault isolation in the PVM is not possible. This problem in all types of PVIs can be decisive to know on which panel to act immediately with regard to its maintenance, in order to disconnect the affected panel, improve the overall performance, avoid catastrophic failure, or when the problem requires additional localized panel analysis using redundant diagnosis methods.

One of the main contributions provided by the approach of the predictive diagnosis method specified in the present work is that it is centred and focused on the PVM, where the degradation of the process mainly takes place. Therefore, the quick detection and isolation in incipient phases are systematically guaranteed. It is online and real-time, using observable electrical parameters of easy accessibility and reliability and allows, to incorporate the possibility of automating actions on the system aimed at avoiding the future failure event, detecting not faults but predictive symptoms.

However, as with other types of industrial facilities, in the third phase of analysis associated with the general concept of fault diagnosis together with the detection and isolation phases, to achieve greater precision in the diagnosis of the type of fault, it is required to use other kinds of techniques and methodologies that provide redundancy, diversity, or greater adaptation to analyse different types of failures. In this sense, methods based on infrared (IR) and electroluminescence (EL) thermography have been used effectively. However, these techniques are not without problems as they require good technical training for the maintenance teams involved [13]. They also present notable differences in the quality of the analyses depending on whether they are carried out in the outdoor or indoor environment and also restrictions to immediate detection when used manually and causing interruptions in normal operation [14]. The use of remotely piloted aircrafts RPA requires non-negligible periods of recharging their batteries [15], their effective use also requires special technical training [16,17], and the equipment is more expensive, cannot compete in terms of speed of detection and isolation with the online predictive diagnosis method based on the use of the electrical parameters of the PVMs.

In a generation system based on photovoltaic solar energy, the PVMs can be connected in series or in parallel. The appearance of defects in a cell will affect the performance of the PVM, and in the case of PVM strings, affects globally the respective string, which will decrease the performance of the photovoltaic system [14,18].

Therefore, in the authors’ opinion, a centralized predictive diagnosis and supervision system based on the use of I-V predictive parameters, monitored in the form of time series prone to the treatment of statistical data and allows the design and implementation of intelligent algorithms, is capable of carrying out immediately and simultaneously two of the three recognized objectives to be carried out by the diagnosis of faults, such as the detection and isolation of the fault centred on the PVM, which is indisputably the fundamental basic unit where to apply maintenance tasks and in a complementary way, immediately apply security actions, such as:

(a) Automatically disconnect the detected and isolated photovoltaic panel to avoid the cumulative effect of degradation and the development of irreversible catastrophic failure.
(b) Optimizing the overall performance of the photovoltaic system in terms of its energy generation objective, taking into account that the operation of a faulty PVM together with a non-faulty PVM causes a greater loss of power generation if they continue to be connected together.

Therefore, in the authors’ opinion, since the first fundamental objective of safeguarding the affected panel has been achieved, based on the use of I-V parameters and their capacity for rapid detection and isolation, taking into account that they are involved in most types of specific PVMs failures and degradation, it is possible with a greater margin of manoeuvre, also in better conditions of safety and energy efficiency of the solar installation, to apply a phase of analysis of the root cause of the degradation process, using complementary redundant methods more complex and deeper, but slower in its application and obtaining results, which allow establishing additional correlations with thermographic and artificial vision parameters for the specific analysis of a great diversity of degradation causes, not only with regard to hot spots but also to the structural physical condition of the MVPs. In this sense, below, a set of references are highlighted whose common denominator is the use of RGB and IRT cameras using various techniques for specific analysis.

Ref. [19] proposes infrared thermography (IRT) as the best technique to identify faults, including hot spot development. Ref. [20], presents a thermal model to simulate the thermal performance of PV modules. This model is coupled with an electrical model and a radiation model to evaluate the electrical performance of the PV panels. Ref. [21] analyses field-aged modules operating for 18–22 years. Degradation effects are observed in severely EVA discoloured PV cells. Temperature degradation effects are identified through IRT in bus bars, contact solder bonds, blisters, hot spots, and hot areas. Agreement between the source of electrical performance degradation and the degradation effects in the defected cell identified by the IRT was found using the I-V curve analysis. Ref. [22] presents Day Light Luminescence System Testing (DaySy), which generates electro- and photoluminescence images of installed solar modules in bright daylight. This analysis easily reveals broken solar cells with interrupted interconnects or cracks. Ref. [23], proposes the use of standard thermal image processing and the Canny edge detection operator as diagnosis tools for module-related faults that lead to hot spot heating effects. These techniques were used on thermal images of defective PV modules from several field infrared thermographic measurements.

However, although manual and ground procedures for the specific diagnosis of the type of failure have been used extensively, they have drawbacks and difficulties, especially for PVIs of large extensions, as they have to be applied in complex conditions and require time in their proper task development [24]. In [25], the automatic supervision and fault detection procedure for PV systems is based on power losses analysis, comparing the thermal capture losses and the miscellaneous capture losses. Ref. [17] presents a non-invasive inspection method providing information of possible failures of photovoltaic modules. This method relates the thermal behaviour of the modules to the operational status of PVMs, monitoring RGB (red, green, and blue) and IRT values. An adequate thermal measurement module strongly depends on the proper camera angle selection relative to the panel’s surface since reflections and external radiation sources are common causes of misleading results with the unnecessary maintenance work [26].

In this context, the use of drones makes it possible to implement different configurations in order to detect different types of failures. One of the most used configurations is the dual configuration formed by an RGB camera and an IRT camera [27]. In [19,28,29,30,31,32], works are cited and described in which many previously commented drawbacks associated with the manual use of thermographic and artificial vision techniques in PVIs have been improved and avoided. This new approach, after the manual use of drones, introduces new advances that increase the automation of diagnosis tasks by applying algorithms for planning drone flight routes, with more precise positioning, systematically applying the correct camera angles regarding the position of the panels obtaining more precise diagnosis data [28], and finding more exact locations of the defective PVMs among hundreds or thousands of PVMs in large PVIs.

The main method of preventing the appearance of hot spots has been the passive bypass diodes placed in parallel with the strings of photovoltaic cells, which is a standard practice used in the manufacture of photovoltaic panels [33,34], to avoid degradant damage that can occur in photovoltaic cells strings [35]. Bypass diodes function as an alternate current path that prevents extreme reverse voltage bias in PV strings. A common misconception is that bypassing a string protects cells from hot spots, but in studies carried out in [36], it is shown that although the problem is mitigated, it does not prevent the appearance of damage caused by hot spots.

In [37,38], another of the solutions proposed to avoid the problem of hot spots is to increase the number of bypass diodes, even up to one for each cell. However, this proposal has not been widely accepted among PVM manufacturers due to the increased cost due to diodes and harmful from the point of view of electrical energy production [39].

A bypass circuit to improve the behaviour of MVPs in the event of hot spots is presented in [40]. A series-connected power MOSFET is used in the design that reduces the reverse voltage of the shaded solar cell. In [41,42], the problems associated with the use of the standard bypass diode are discussed, and its replacement by a switch with a single board design and control logic is proposed, but its practical application is debatable. The design and development of hot spot mitigation techniques using a simple, costless, and reliable method are proposed in [43,44]. The hot spots analysis in the PV system was carried out using a FLIER i5 IRT.

On the other hand, in recent years, PVM optimizer devices have appeared that are presented as the solution to the problem of hot spots, and more importantly, Solar Power Optimizers break the trend of focusing monitoring and diagnostics exclusively on the inverter of PVMs strings since they allow supervision and diagnosis on each of the PVMs. But their price still does not make their widespread implementation easy. An alternative for the generalization of supervision at the PVM level can be in low-cost IoT sensors, not losing sight of their reliability [45,46,47].

The MPPT solar regulation approach proposed in [45] mitigates the hot spot in partially shaded (small) PVMs with a temperature reduction of up to 20 °C, using a drive parameter selection optimization procedure and a slider algorithm, using an MPPT approach with perturbation and observation (P&O) algorithm to track the point of maximum real power through successive approximations. In [48], a proposal is made for the optimal location of voltage sensors for online fault diagnosis of a photovoltaic array, with the objective of reducing maintenance costs.

Ref. [49] presents an Internet of Things Technology (IoT) proposal for historical analysis of a PVM and also for real-time remotely monitoring, performance evaluation, preventive maintenance, and fault detection.

Similar IoT based cases can be found in [50,51], including low cost embedded solar PV monitoring system, GPRS module to send data via the internet and global accessing, providing real-time information on help maintenance and fault diagnosis, in the first and including smart sensors, a cutting-edge controller and an algorithm for solar array monitoring integrating alerts for anomaly detections in PV stations, in the second.

Due to its importance, several methods are being developed to carry out the supervision of solar panels in real-time [52,53]. These methods propose the use of wireless sensor networks and communication through low-range Zigbee, sending current, voltage, radiation, and temperature data to monitor the solar panels’ parameter curves in a web or mobile application. It should be noted that these devices have little wireless connection range. In [54], a method is proposed for the acquisition of data on solar irradiance, environmental temperature, wind speed and direction, voltage, current, and panel temperatures through a PcDuino. This method allows the diagnosis of faults in real-time, saving the data to an SD card. This method isolates the failed solar panel for maintenance. However, it is not possible to control every solar panel on the electrical network to which they are attached. The methods proposed in [55,56] achieve the solar panels’ fault diagnosis using data from current, voltage and other meteorological sensors using various platforms such as Arduino and Raspberry Pi for data acquisition. They are sent over the internet to a server for later representation on a web page, achieving supervision. However, their overall cost is close to 100 euros, still being a high value to be implemented in each solar panel. Most of the works focus on the I-V curves, as in [57] that proposes the acquisition of data with the Arduino board. The sensors used to measure the current is ACS712 with hall effect, and for the voltage, a divider voltage sensor allows to monitor Isc and Voc, as well as I-V with variable load. It only shows the measurements in the Arduino serial monitor, and the data is plotted in Excel. Therefore, this type of supervision is not recommended because it cannot be done in real-time, and the Arduino board must always be connected to the computer (PC).

In [58,59], another method for panel diagnosis is indicated, and it presents remote mode monitoring with Arduino for data acquisition and for sending the data to the ThinkSpeak server with the ESP8266 board. This server allows viewing on a dashboard the data collected with the ACS712-30A sensors and a voltage sensor to acquire the I-V data. Also, in [60], the data is saved in the ADAFRUIT CLOUD server, where they are displayed in a dashboard offered by the server. It should be noted that these servers are not free, which means an additional cost, and the solar panels can only be viewed but not controlled. In [61], a new method is proposed to design radiofrequency antennas in the crystals of solar panels working as cheap sensor transponders, performing a better fault diagnosis in the I-V curves. However, this new technique does not allow to operate with the solar panels being not very efficient in case of serious failures.

Within this order of ideas, [62] shows the supervision and operation of solar panels using the fuzzy NARX neural network fault arrest technique with IoT (Internet of Things) technology. This technique detects the fault quickly and accurately, even achieving real-time operation, which means that the solar panel can be disconnected from the system in case of failing. Although this is a great advance from the point of view of automatic control supervision, it should be noted that this method only allows the disconnection of the solar panels in pairs, causing a disadvantage when optimizing their production in case of solar panel faults. In [63], a method for monitoring with an open-source platform based on IoT is proposed with the advantages it has over SCADA systems using the Eclipse Kura and Eclipse Kapua software to transmit data in real-time or in batches, as well as hardware necessary for the gateway (Kura) and the server (Kapua), uses PC Moxa UC-2112 which transmits the data through any of the communication protocols such as TCP, OPC, Modbus TCP/IP, MQTT, OPC-UA. These protocols are also used in some research [64,65,66]. However, the communication is executed by MQTT from the Kapua server, sending the data from each of the solar panels to be displayed on the Grafana platform, where the drawn curves correspond to the parameters measured in the solar panels. Although the method is very innovative in terms of new communication technologies, it has a gap in automatic operation. It is also necessary to indicate that Eclipse Kura is a non-standardized version, which is why some stability problems arise with various hardware devices.

As technology advances, new proposals continue to emerge, such as in [59] where it is proposed to carry out tests in situ to diagnose faults in individual solar panels, based on the use of a device called SmartPV with a cost of around 40 euros. This device allows obtaining the operating parameters of each solar panel such as voltage, current, temperature, the intensity of solar radiation, ambient temperature, and humidity. The data transmission is done by wireless to a server, but the data only allows to visualize the curves of each one of the parameters. Hence it does not allow any action to be carried out on the solar panel, such as the automatic disconnection of a string from the solar panels, showing a disadvantage in case of a serious failure that puts a large part of the production at risk, which is why there is no progress in terms of automatic control.

In this work, the proposed predictive fault diagnosis method is tested using an experimental test bench for the operation of three solar panels connected in series, on which the automatic disconnection of the solar panel can be performed in real-time if a predictive symptom of a possible future failure is detected. The development of this methodology is done with the use of the ESP8266 module. The supervision and control of the solar panels are carried out in the HMI/SCADA software (iFIX 6.5) dedicated to the automation of the GE (General Electric) family of products. It is reliable industrial software with many years of implantation in the industrial market. The work carried out shows that low-cost technology can be integrated with robust industrial software without the need for a PLC, making the application of low-cost predictive fault diagnosis compatible. This method shows the supervision of the three solar panels in real-time, being able to perform tests on the three solar panels, based on the disconnection of each of the solar panels to perform Voc-Isc tests looking for possible downward deviations of the parameters as predictive symptoms. The test makes it possible to detect if the solar panels are developing in an initial stage the degrading phenomenon that will cumulatively lead in the near future to a non-recoverable failure. Consequently, it is able to isolate the faulty solar panel and perform the permanent automatic disconnection so that it does not harm the power generation of the complete string while the pertinent maintenance actions are taken.

## 3. Materials and Methods

This part of the work is divided into two sections: The first section exposes the hardware and software tools used, and the second section explains the applied methodology.

### 3.1. Materials

The materials used in the SCADA system of three solar panels connected in a string for the predictive diagnosis of failures (Automatic Monitoring and Control) by means of tests of voltage in open circuit and current in short circuit in each of the solar panels that make up the system using the ESP8266 module are described below.

#### 3.1.1. Solar Panels

**Figure 1:**
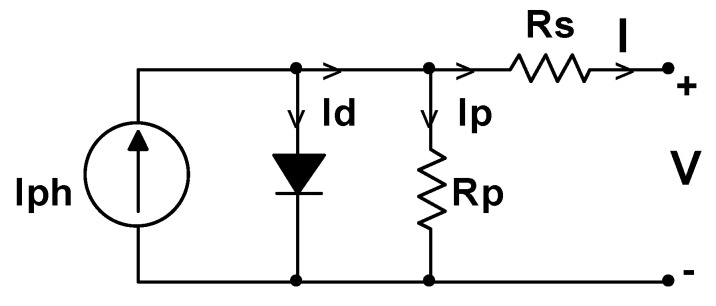
Basic circuit of a solar cell.

Solar panels are composed of an arrangement of solar cells that convert solar irradiance to electrical energy, basically constituting an n and p-type silicon semiconductor. The basic circuit that defines a solar cell is shown in Figure 1, where it can be seen that the model consists of a single diode and four elements such as a current source, a diode, a resistance in parallel to the source and the diode, and a series resistance [67,68,69,70,71].

**Figure 2:**
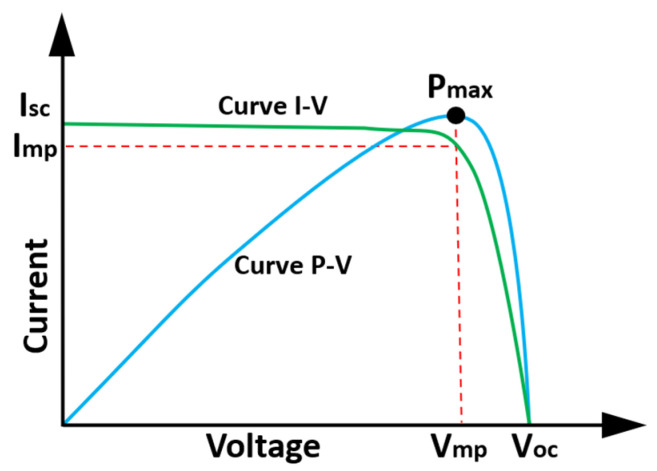
The I-V and P-V curves of a photovoltaic device (Adapted with permission from ref. [72]. Copyright 2015 Kumar, P., et al.).

The equations to calculate the current and the voltage that defines the curves of the solar cell in Figure 1 can be seen in [67,68,70]. Figure 2 shows the curves of current, voltage and power, under conditions of solar radiation of 1000 W/m^2^ and a temperature of 25 °C, which are the standard parameters under optimal operating conditions. It should also be taken into account that the output power is directly proportional to the aforementioned parameters (solar irradiance and temperature). The output power is conditioned by several parameters that can affect its performance, for example, partial, or total shading, in addition to some failure in the solar panels described previously.

**Figure 3:**
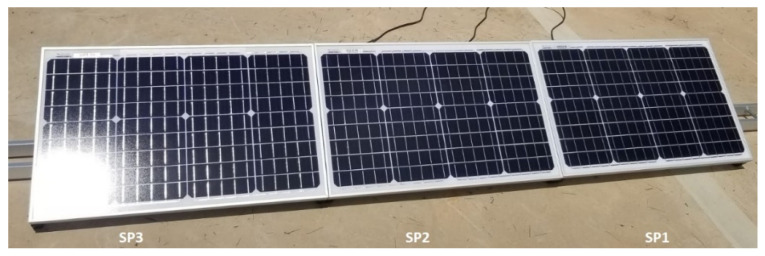
View of solar panels in String.

In this work, three monocrystalline solar panels of the Victron Energy Blue Power brand of 40 W were used (Figure 3). Their characteristics are described in Table 1. They are manufactured with high quality and high transmission tempered glass for better toughness and impact resistance. They also have a solid galvanized aluminium frame for installation in different mounting systems. Its connection box is hermetic, providing high security. There is a bypass diode for the automatic disconnection of cells affected by a shading event. The output power will always depend on the standard parameters described in Table 1 [73].

#### 3.1.2. ESP8266 Module

The ESP8266 device was developed by the company Espressif and is characterized by being a low-cost chip that is used in conjunction with the NodeMCU module with a wireless internet connection. The operating voltage is 3.3 V; however, it allows input up to 10 V and 80 mA of current, its processing speed is 160 Mhz. Apart from being a low-cost device, it is also energy-efficient and easy to program. The module is composed of several pins such as GPIO, SDIO, SPI/HSPI, I2C, I2S, UART, PWM, IR, and ADC. There are several versions of this module [74].

This device is widely used today for its versatility in the development of applications that need to connect to the internet to transmit data or operations in real-time. It is also used in IoT applications. There are also several investigations in which they have worked with this technology in different scientific areas [75,76,77,78,79].

**Figure 4:**
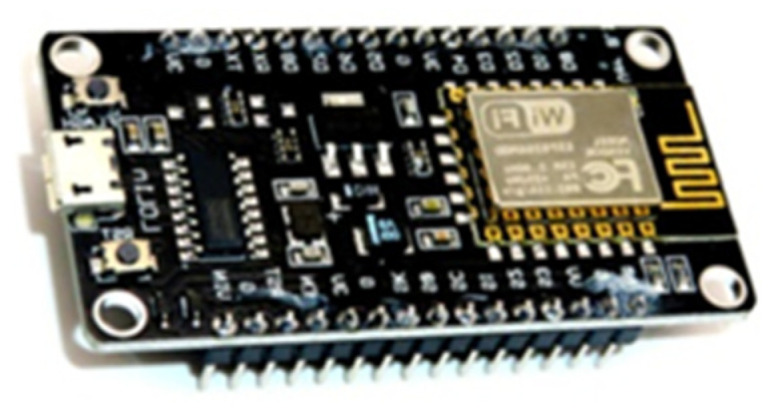
NodeMCU V3 CH340 ESP8266 module.

In this work, a NodeMCU V3 CH340 ESP8266 module is used that allows to acquire data and control the connection and disconnection of the solar panels in real-time (Figure 4).

The disadvantage of the device is that it only has one analogue input pin (DCA), an important feature to be able to use the module when there is more than one analogue input. For this reason, in this work, a multiplexer/demultiplexer that is described below has been used.

#### 3.1.3. CD74HC4067 Module

**Figure 5:**
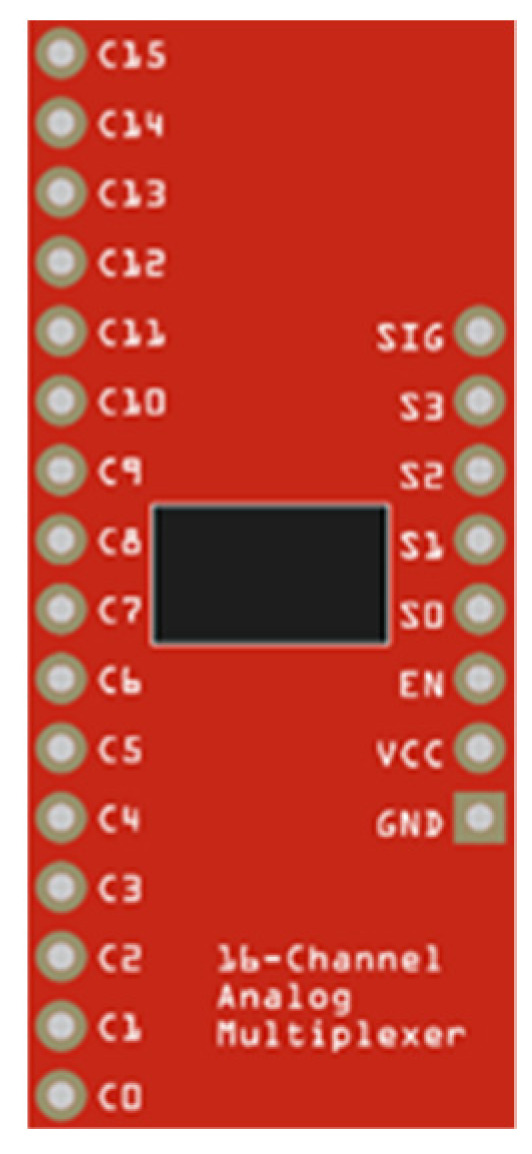
Modulo CD74HC4067.

This module allows increasing the analogue inputs in ESP8266. The module works with a voltage range from 2 V to 6 V. For its configuration, four digital inputs, two GND (ground) inputs, an analogue input, and Vcc (Direct current voltage), are needed (Figure 5) [80]. Its input pins are defined by the configuration described in Table 2. This Table is created from the four digital input pins (4 pins of the ESP8266), creating a matrix with 16 input channels that transmit their data to the digital analogue input of the ESP8266.

#### 3.1.4. ACS712 Current Sensor

ACS712 is a Hall-type sensor that measures the intensity of both alternating and direct current that passes through the conductor. Its operation is through the Hall effect; that is, it measures the current when the magnetic field is perpendicular to the conductor, generating a voltage difference proportional to the current that passes through it. This sensor is widely used when working with microcontrollers such as Arduino, Raspberry Pi or, in this case, ESP8266. This sensor works with 5 V; the variation of the amperage depends on the selected model that can be 5 A, 20 A, 30 A (Table 3). It should be borne in mind that this sensor cannot be used if there is a very strong magnetic field because it would affect the reading resolution [81,82,83].

**Figure 6:**
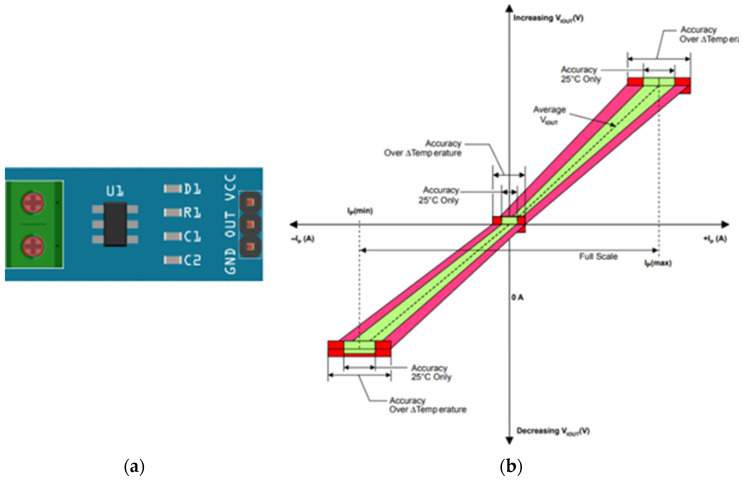
Current Sensor; (**a**) current sensor; (**b**) current sensor operating curve.

The sensor for this work is implemented in a module that allows making the connections easily (Figure 6a). The operating curve (Figure 6b) shows that the average voltage at the sensor output is 2.5 V indicating that the current is 0 A. This value varies as indicated above. The nominal current of the sensor used in this work is 5 A, and its sensitivity is 185 mV/A. If the voltage is less than 2.5 V, it means that the current is negative.

#### 3.1.5. Voltage Sensor FZ0430

**Figure 7:**
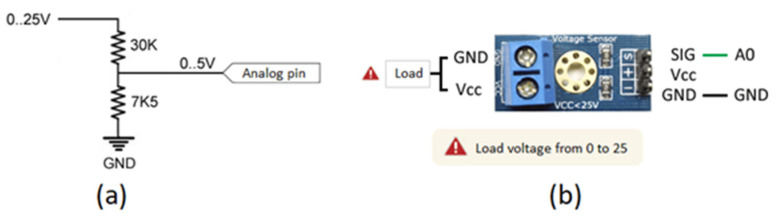
Voltage Sensor; (**a**) Voltage Divider; (**b**) Voltage Sensor Module.

The FZ0430 voltage sensor consists of a voltage divider (Figure 7) and has a resolution of 24.41 mV in its measurement. This sensor can measure up to a maximum of 25 V in microcontrollers that work with 5 V. On the other hand, in microcontrollers that work with 3.3 V, such as the ESP8266 module, it is possible to read voltages up to 16.5 V [84].

In this work, the readings to be measured are higher than 16.5 V according to the characteristics of the solar panel (Table 1), this being a disadvantage when using this sensor. However, being a voltage divider sensor inside, it is easy to modify the input. It simply requires adding a higher resistance to extend the voltage range at the input. The calculations are performed later in Section 3.2.

#### 3.1.6. Relay Modules

**Figure 8:**
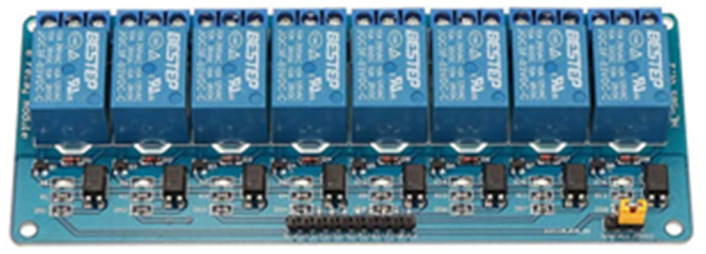
8-channel relay module.

The relay modules allow the switching of loads through mechanical action. The module is made up of optocouplers to connect and disconnect the loads. The relays are activated when it exceeds their operating value, and the control can be done from microcontrollers, Arduino, ESP8266, etc. In addition, each relay is composed of COM, NO, and NC pins, and its operating voltage depends on its characteristics. In this work, the 8-channel module is used, and its operating characteristics are in DC 30 V/10 A and AC 250 V/10 A, and the activation voltage is 3.3 V (Figure 8) [85].

#### 3.1.7. Software

In the development of this work, various software programs were used for communication, data acquisition and integration with the SCADA system. Their joint use has made it possible to achieve the supervision, predictive diagnosis, and real-time operation of the solar panels, carrying out the integration of low-cost technology with industrial software of proven quality. The integration carried out is described in Table 4.

### 3.2. Methodology

Online supervision and control of solar panels are of great importance to apply predictive fault diagnosis. For this reason, in this work, an easy and fast solution is proposed, with the low-cost sensors, devices, and technology mentioned in Section 3.1. In addition, communication is performed with the software described in the previous section, as well as the development of the algorithm on the SCADA System.

**Figure 9:**
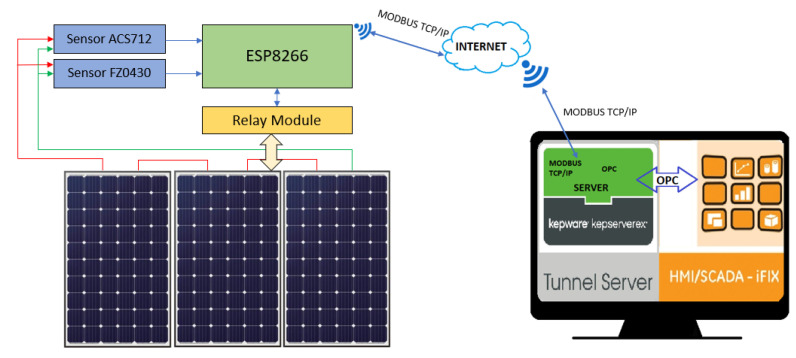
SCADA architecture.

The ESP8266 module reads the data from the sensors and also allows the operation of connection/disconnection of the solar panels. This data is sent to the internet through the Modbus TCP/IP communication protocol to a local computer in which Kepserver and iFIX software are installed. Kepserver software is configured with the same communication protocol as ESP8266, and it should be noted that Kepserver software allows the configuration of various communication protocols in a versatile way. Additionally, Kepserver software acts as a communication broker with iFIX via local OPC communication. In iFIX, the data that has been transmitted from the ESP8266 module is read and displayed on the SCADA system dashboards. Here the connection/disconnection operation of the solar panels is also performed. The development architecture of the proposed model is described below (Figure 9).

**Figure 10:**
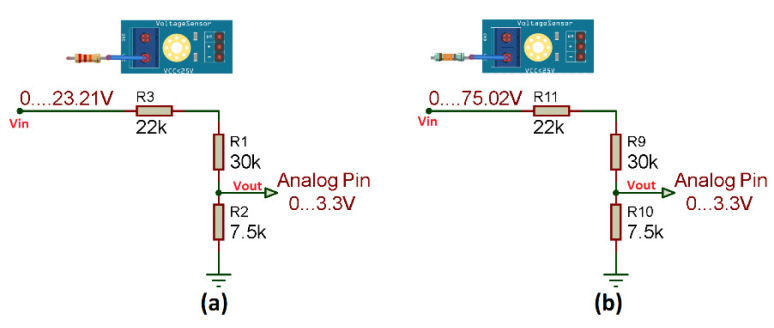
Modification of voltage sensors; (**a**) Sensor for the Voc test of solar panel; (**b**) Voltage sensor Voc of solar panels string.

As explained in Section 3.1.5, the ESP8266 module only allows to measure voltages from 0 to 16.5 V. However, the voltage measurement range can be extended, adding a new resistance at the V+ input. This change is made in the two sensors, both for the solar panel string sensor and on the sensor for the Voc test of each solar panel (Figure 10).

**Figure 11:**
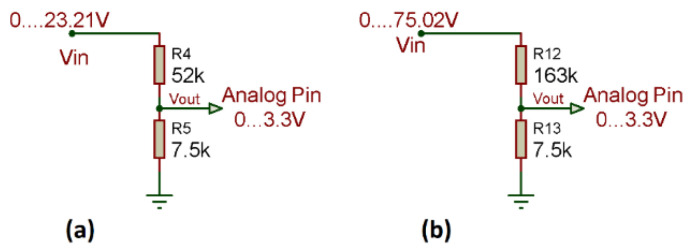
New voltage sensors; (**a**) New sensor for the Voc test of solar panel; (**b**) New voltage sensor Voc of solar panels string.

The voltage value that the ESP8266 module can read must be calculated, but first the calculation of the new resistance must be performed (Figure 11) as described in Equation (1).

(1) $$\begin{array}{l} RT=R1+R3\;\;\;RT=R4+R6\\ RT=30\;k\Omega +22\;k\Omega \;RT=30\;k\Omega +133\;k\Omega \\ RT=52\;k\Omega \;\;\;\;RT=163\;k\Omega \end{array}$$

Once the new resistance value has been obtained, it is necessary to know the value that the sensor can measure from the ESP8266 module according to Equation (2).

(2) $$\begin{array}{l} V_{out}=V_{in}\frac{R5}{\left(R4+R5\right)}\;\;\;V_{out}=V_{in}\frac{R13}{\left(R12+R13\right)}\\ V_{in}=V_{out}\frac{\left(R4+R5\right)}{R5}\;\;\;V_{in}=V_{out}\frac{\left(R12+R13\right)}{R13}\\ V_{in}=3.3\;V\frac{\left(52\;K\Omega +7.5\;k\Omega \right)}{7.5\;k\Omega }\;\;V_{in}=3.3\;V\frac{\left(163\;K\Omega +7.5\;k\Omega \right)}{7.5\;k\Omega }\\ V_{in}=23.21\;V\;\;\;\;V_{in}=75.02\;V \end{array}$$

**Figure 12:**
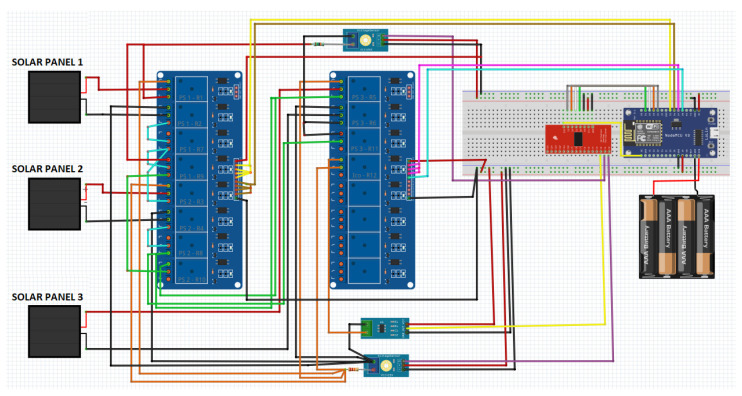
Solar panels monitoring and control circuit.

From the calculations and the changes made in the sensors previously, the circuit is designed for both the data reading and the operation of the solar panels, being necessary to make the connections shown in Figure 12.

Additionally, it must be indicated that the resolution of the ESP8266 module is 10 bits. This means that a voltage from 0 V to 3.3 V is represented from 0 to 1024. On the other hand, it is important to mention that for the acquisition of data from the sensors is necessary to program the ESP8266 module, whose programming is carried out in the Arduino IDE software, being essential to add several libraries for its correct operation.

After the data has been read from the voltage and current sensors by the ESP8266 module, it is sent through the Modbus TCP/IP communication protocol to the remote computer where the iFIX and KepserverEX software are installed. The KepserverEX software is used as a communication tunnel between the ESP8622 module and the SCADA (iFIX). It is important to point out that for communication between the aforementioned software is necessary to configure the OPC communication protocol in iFIX.

**Figure 13:**
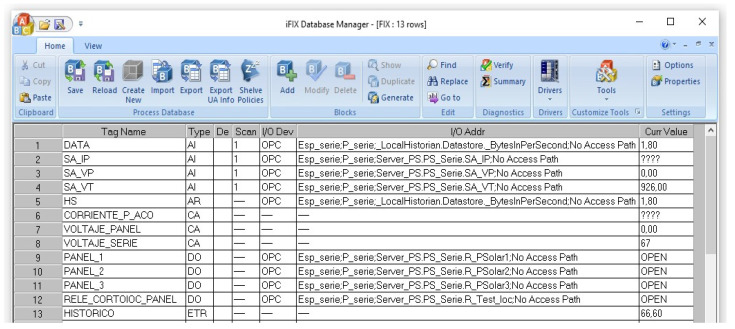
Creation of tags in iFIX 6.5.

Data transferred from KepserverEx to iFIX is defined by analogue signal and digital signal tags (Figure 13). Furthermore, the calculations to indicate the values read by the current and voltage sensors are performed in calculation tags. It is also convenient to note that the ACS712 current sensor must be powered at 5 V (2.5 V being equivalent to 0 A). It must be taken into account that the reference voltage of the ESP8266 module is 3.3 V. Therefore, calculations must be made to show the correct reading (Equations (3)–(5)).

(3) $$V_{in}>2.5\to +↑A$$

(4) $$V_{in}=2.5\to \;0\;A$$

(5) $$V_{in}<2.5\to -↓A$$

The value of the current (I) measured by the sensor is defined by Equation (7), where *Vin* is the voltage that enters pin 2 of the CD74HC4067 module (ACS712 sensor output pin), the resolution is the voltage of the ESP8266 reference (3.3 V) for 10 bits (1024), and the sensitivity of the ACS712-5A sensor is 0.185 μV/A (Equations (7) and (8)).

(6) Sensor sensitivity=0.185 μV →ACS712(5A)

(7) Reference voltage resolution=3.3 V/1024

(8) $$I=\left\{\left[\left(V_{in}*Resolution\right)-2.5\;V\right]/Sensibility\right\}\;A$$

On the other hand, the voltage read by the ESP8266 module is interpreted according to Equation (9). Calculations are performed in a calculation tag within the iFIX software [86].

(9) $$V=\left(V_{in}-R_{min}\right)*\left(\frac{V_{max}-V_{min}}{R_{max}-R_{min}}\right)+V_{min}$$

**Figure 14:**
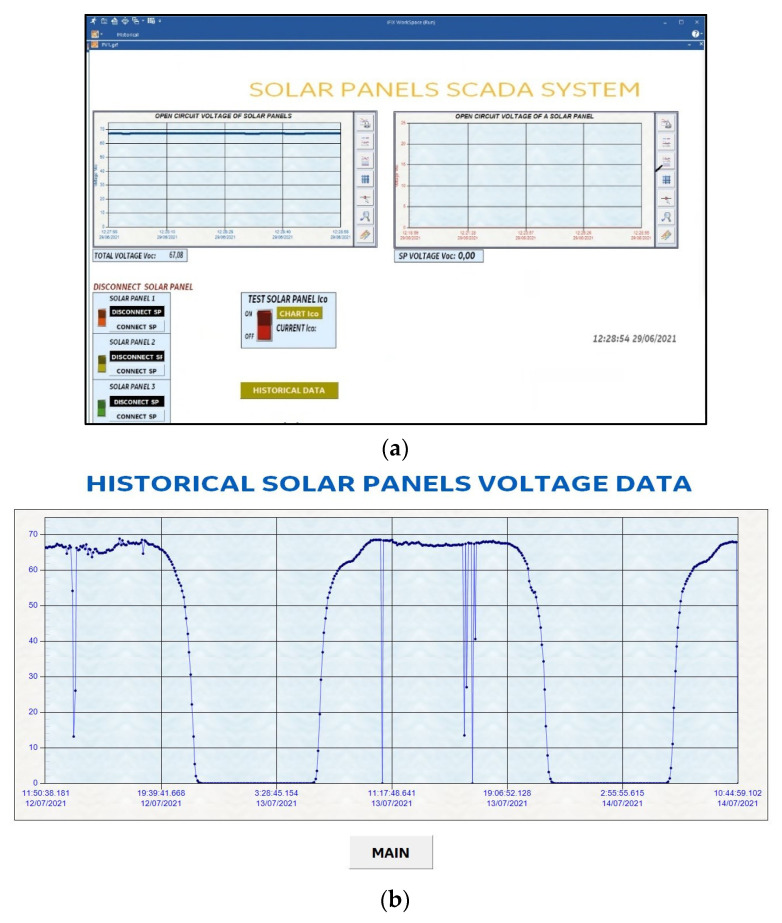
SCADA system; (**a**) Main screen; (**b**) Historical data screen.

$V_{in}$ is the voltage that enters pin 0 and 1 of the CD74HC4067 module, *R_min_* is the minimum resolution of the ESP8266 module (*R_min_* = 0); *V_max_* is the maximum voltage from Equation 2 (*V_max_* = 23.21 V or 75.05 V), and *R_max_* is the maximum resolution of the ESP8266 module (*R_max_* = 1024). The calculated values of the voltage and current sensors are shown on the SCADA dashboard made in iFIX (Figure 14).

The Voc-Isc tests are carried out through short-term programmed serial disconnections of each solar panel to graph its curves on the SCADA dashboard. The fault diagnosis method looks for possible comparative downward deviations of the parameters as predictive symptoms indicative that a fault is developing in an initial stage. This degrading phenomenon will cumulatively lead in the near future to a non-recoverable failure. It is necessary to indicate that the Voc test is carried out first, and then the Isc test is carried out. This same process is repeated for all the solar panels connected to the string.

#### Predictive Fault Diagnosis Method

Predictive fault diagnosis is based on carrying out experimental tests to locate predictive symptoms. The Voc-Isc curves are plotted on the same dashboard to facilitate the online comparison of the curves of the three solar panels.

The data update can be done every 100 ms, which is the recommended or standard update defined by KEPServerEX V6; however, this can be defined by the user, depending on the type of application and the characteristics of the hardware that is being attached to the software. In this case, a period of 1 s was used, which is enough to transfer data because programming the ESP8266 module, a sample of 300 readings is made, which are averaged to have a single reading and sent every second thus giving a more reliable result. In the KEPServerEX V6 software configuration, a time of 1 s to wait for the update is defined.

**Figure 15:**
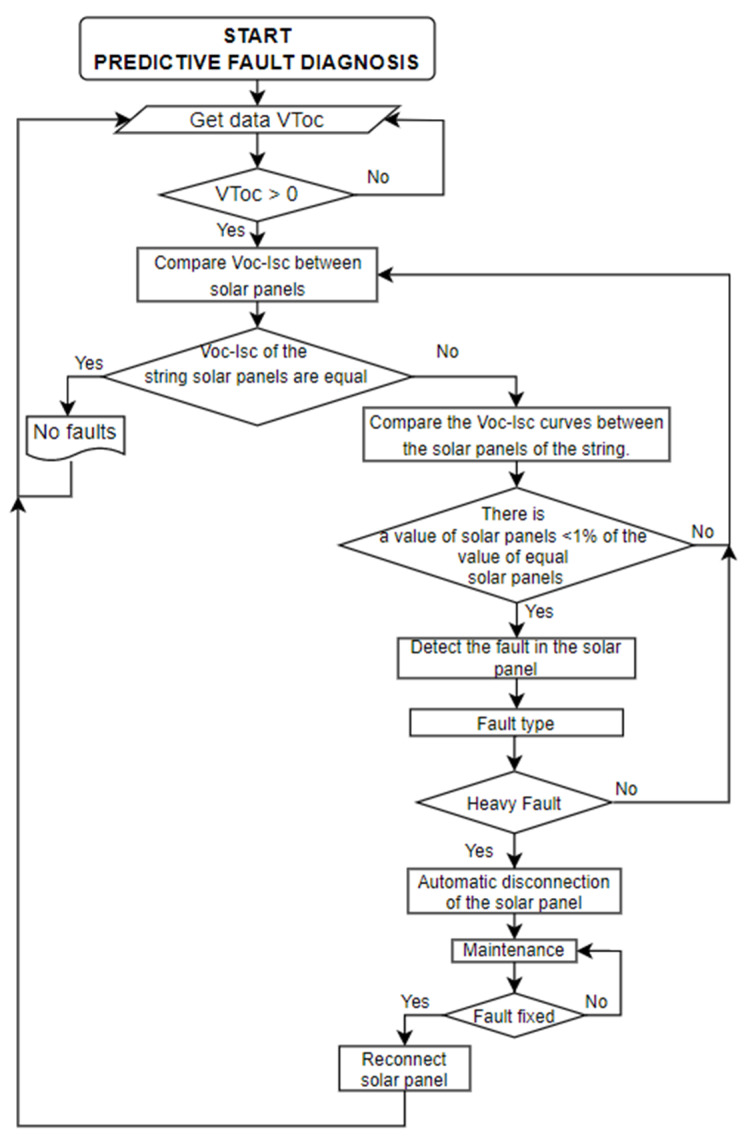
Flow chart for predictive fault diagnosis.

The Voc-Isc curves must be similar in normal conditions because they are from the same type of PVMs with the same characteristics. However, if any of them is different, it can be understood that a fault symptom has occurred and has been detected. For every fault symptom, it is necessary to automatically isolate the solar panel so as not to compromise the entire production of the solar panel string until analysing the type of fault, the cause and finally giving it the necessary attention to avoid the failure, and subsequently reconnect it to the string of solar panels. In this way, it is detected a symptom that can subsequently generate a hot spot or irreparable damage to the solar panels (Figure 15).

Finally, the proposed method for parametric predictive fault diagnosis is verified through 8 experiments. In the first experiment, the solar panels are operating correctly and without shadows, and the remaining seven were carried out covering different areas of the solar panel, representing partial shadowing. The following section explains the results of the experiments.

## 4. Results and Discussion

This section presents the different experiments carried out in the first solar panel (SP1) (Figure 3). These experiments are based firstly on the string of solar panels without shadows and then covering different areas of the solar panel to observe the behaviour of the Isc-Voc curve with respect to the other solar panels. The experiment was carried out on the terrace of Building 5C of the Polytechnic University of Valencia (latitude and longitude: +39°28′56.53′′, −0° 20′36.88′′). Table 1 shows that the dimensions of the solar module are 37 × 63.5 cm, and its area is 0.235 m^2^.

### 4.1. Sensor Measurement Check

The calibration of the sensors is important to know the measurement error of the sensor with respect to the DT-33D multimetre because the reading of the sensors must comply with the IEC61724 standard. The standard indicates that the accuracy of the measurement in voltage and current must have a maximum error of 1%, and the maximum error of the power must be 2% [55].

**Figure 16:**
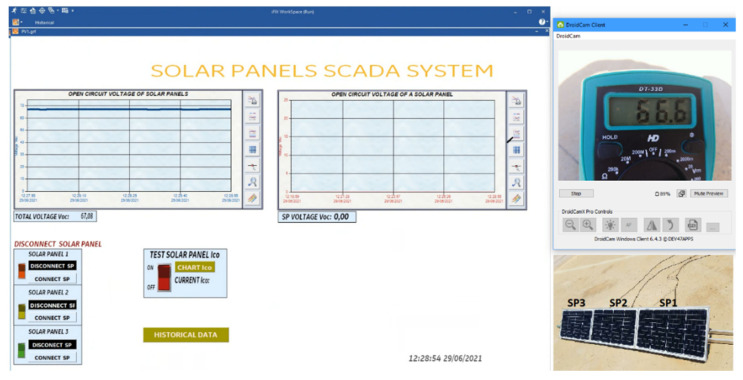
Voc measurement in string solar panels.

**Figure 17:**
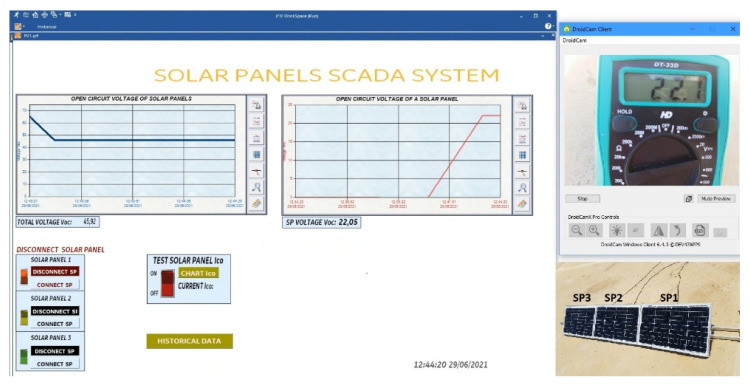
Voc-Isc tests in SP1.

(1) Voc measurement of the solar panel string (Figure 16).
(2) Measurement of Isc and Voc of a solar panel (Figure 17).

The measured data comply with the IEC61724 standard. Table 5 indicates that the voltage sensor reading error is below 1% as required by the standard.

Table 6 shows the measurements made on the three panels by the ACS712 sensor, verifying that the IEC61724 standard is met in the Isc measurement.

### 4.2. Solar Panel Experiments

The experiments in this section consist of the analysis of the behaviour of the curve Isc-Voc for predictive faults diagnosis.

#### 4.2.1. Solar Panels without Shadows

**Figure 18:**
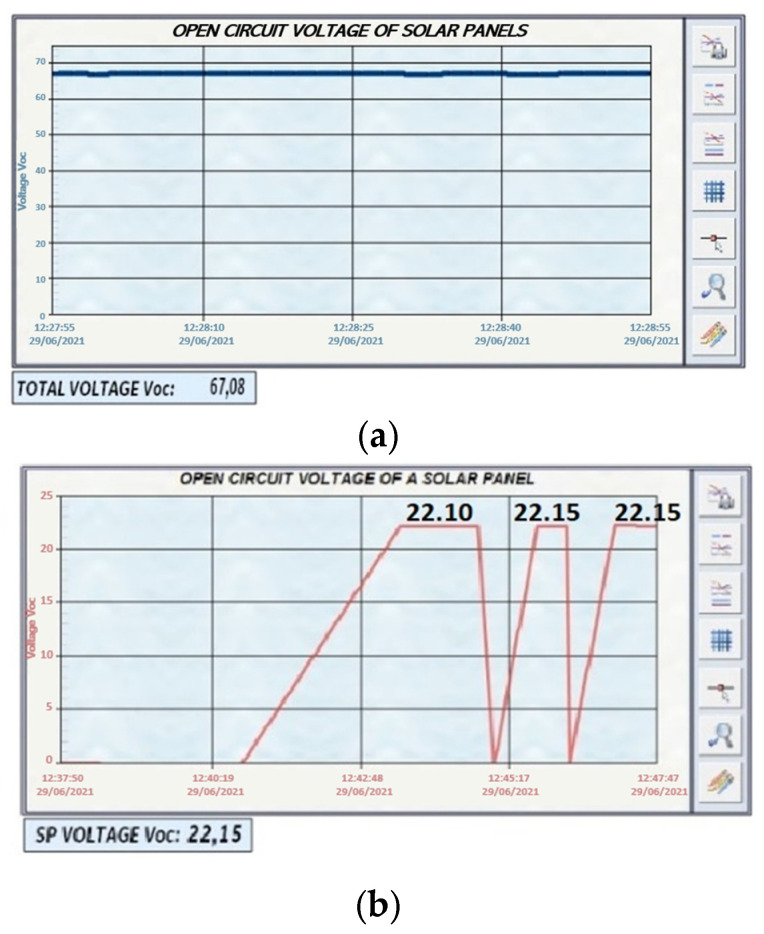

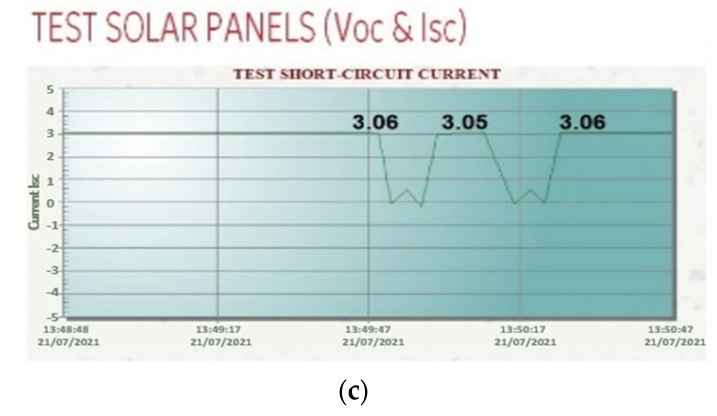
Voc test; (**a**) Voc test to the SP string; (**b**) Voc test to each one of the solar panels; (**c**) Isc test to each of the solar panels.

In Figure 18, the voltage produced by the string of solar panels in open circuit in its normal operation is shown. The Voc curves of each of the solar panels are also plotted. In Figure 18a, the sum of the voltage of the three solar panels connected in string is 67.08 V. Figure 18b has two dashboards. The first dashboard (left) shows that the voltage Voc of the solar panel string decreases when a solar panel is disconnected. The second dashboard (right) shows that the three Voc curves are similar because they are under the same conditions, showing a variation of 0.002257% in one of the solar panels. In Figure 18c, the Isc curves presented are for each solar panel. In addition, they present the same behaviour as in Figure 18b.

The tests carried out on each of the solar panels were performed in real-time. The curves are different if the solar panels are exposed to different conditions or when detecting a fault.

#### 4.2.2. Experiments with Covered Areas of Different Sizes in a Solar Panel

In this section, seven different experiments were carried out on a single solar panel (SP1) of the string to analyse the behaviour of its Isc-Voc curves. The shaded areas used for the experiment are listed in Table 7.

Case (a) 0.80% shadow area

**Figure 19:**
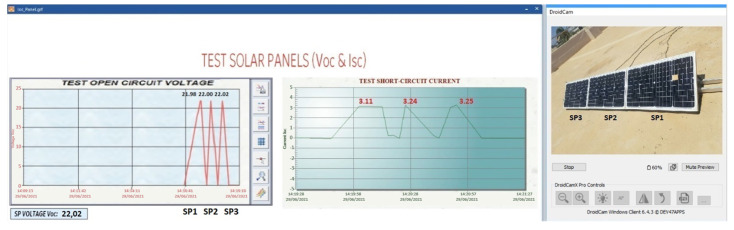
Isc and Voc test at SP1 with a shaded area of 0.8%.

In Figure 19, it can be seen that an area of 0.80% of the SP1 is covered. This represents a cell of the solar panel. When performing the Voc-Isc tests in SP1 and then in the other solar panels, it can be observed that the Isc-Voc curves of SP1 do not show significant changes in Voc with respect to the other solar panels. However, a small impact on the Isc curve of SP1 with respect to the other solar panels can be observed (Figure 19). Although the changes are not significant, it is predicting a fault, which is not significant at the moment, but which may generate a hot spot in the future.

Case (b) 2.52% shadow area

**Figure 20:**
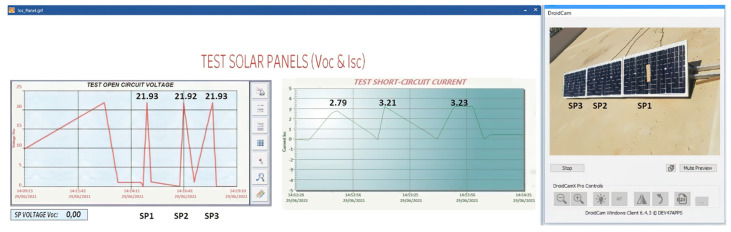
Isc and Voc test at SP1 with a shaded area of 2.52%.

In Figure 20, it can be seen that the shaded area covers 2.52% of SP1, representing four cells of SP1. When performing the Voc-Isc tests, the change in the Voc curve of SP1 does not reflect any change in reference to the Voc of the other solar panels. The Isc curve of SP1 with respect to the other solar panels indicates that there is a short-circuit current drop of 13%, proving that the proposed method detects the shadow fault in SP1.

Case (c) 5.98% shadow area

**Figure 21:**
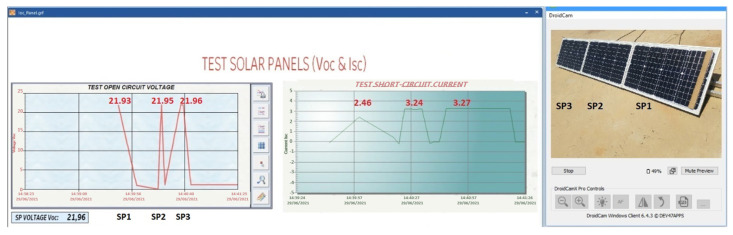
Isc and Voc test at SP1 with shaded area 5.98%.

In Figure 21, the shaded area is increased in relation to the previous experiment. An entire column of SP1 solar cells is covered. As a result, the Voc-Isc tests carried out in SP1 show that in the Voc curve, there is a slight change, decreasing by 0.11% in reference to the other solar panels. The Isc curve of SP1 can be seen to decrease 24% in relation to the other Isc curves. This value is significant, so an important fault has been detected that must be reviewed.

Case (d) 10.73% shadow area

**Figure 22:**
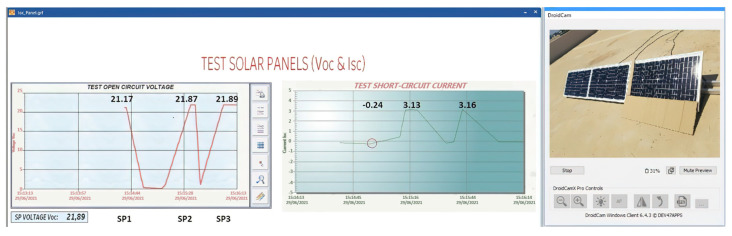
Isc and Voc test at SP1 with shaded area 10.73%.

In Figure 22, a whole row of solar cells is covered, and the Voc-Isc tests are carried out, providing as a result that the Voc is reduced by 0.77% in SP1. This decrease does not present significant changes in relation to the other solar panels. The result of the Isc test in SP1 decreases abruptly by 108% when compared with the Isc of the other solar panels. It can also be observed that the SP1 begins to consume energy from the other solar panels reducing their production. Therefore, this fault has a lot of relevance, significantly affecting production. It is necessary to immediately disconnect SP1 from the solar panel string until proper maintenance is performed. Therefore, it is once again demonstrated that the proposed method is effective and reliable when detecting fault symptoms.

Case (e) Shaded area at 14.22%

**Figure 23:**
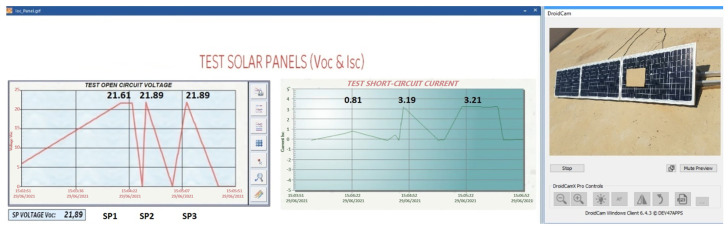
Isc and Voc test at SP1 with shaded area 14.22%.

In Figure 23, the shadow area is greater than in the previous experiment. In the Voc-Isc tests, a decrease of 1.28% is observed in the voltage of the Voc curve of SP1. Isc decreases by 75% in relation to the other solar panels. This decrease is not as serious as in the previous experiment, where SP1 behaved as a load, the shading area being smaller. This fact is due to the configuration of the solar cells. It is still critical as it produces 25% power compared to other solar panels.

Case (f) Shaded area at 26.34%

**Figure 24:**
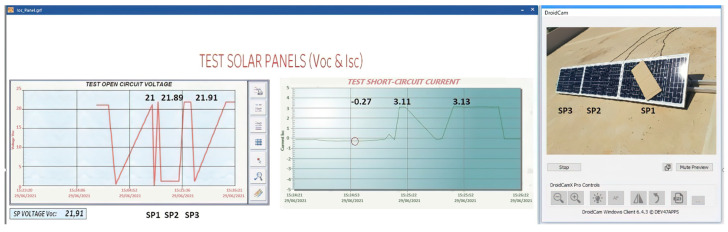
Isc and Voc test at SP1 with shaded area 26.34%.

In Figure 24, the shadow area represents more than a quarter of the solar panel. The Voc-Isc tests carried out show that there is a decrease of 4.11% in the Voc of SP1. Despite this, the difference in Voltage Voc in relation to the other panels is not so great. On the other hand, it can be seen that the Isc of SP1 has a quite abrupt drop and that the panel begins to absorb energy and not to generate current in reference to the other solar panels. Its decrease is 109% being a percentage that compromises all production. Therefore, it must be disconnected from the string of solar panels. The disconnection of SP1 is done automatically and online.

Case (g) Shaded area at 65.00%

**Figure 25:**
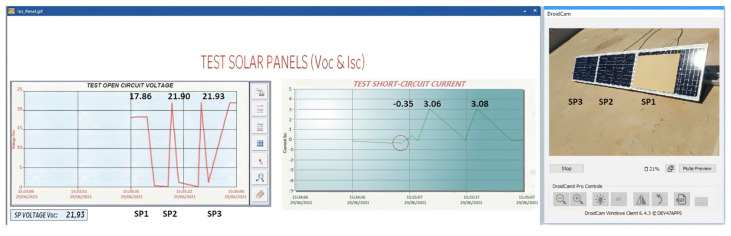
Isc and Voc test at SP1 with 65% shaded area.

In Figure 25, the experiment consists of shading 65% of SP1. Voc of SP1 decreases by 19% in relation to the other solar panels. This time the change in the Voc curve is evident, but it is still capable of producing a high voltage. The same does not happen when the Isc test is performed on each of the solar panels. It can be seen that, once again, the Isc curve of SP1 decreases with respect to the other solar panels by 111.4%. This indicates that the solar panel is consuming current from the other panels.

The results of this experiment show that the SP2 and SP3 panels have reduced their generation by 3.7% and 3.4%, respectively, compared with the results of the first experiment with shadow 1. This implies that SP1 must be disconnected from the solar panel string.

Once again, it is demonstrated that the proposed method is efficient in detecting fault symptoms in real-time and predictively. The panel with the fault symptom can be disconnected without compromising the production of electrical energy from the other solar panels connected in string.

After the experiments carried out, it can be seen that the method developed allows predictive fault diagnosis and also the operation of the solar panels in real-time, guaranteeing that through the Voc-Isc tests, fault symptoms can be detected and also disconnect the solar panel from the solar panel string, without threatening production until proper maintenance is performed and the solar panel is reconnected to the string.

### 4.3. Historical Data of Voc of the String of Solar Panels

**Figure 26:**
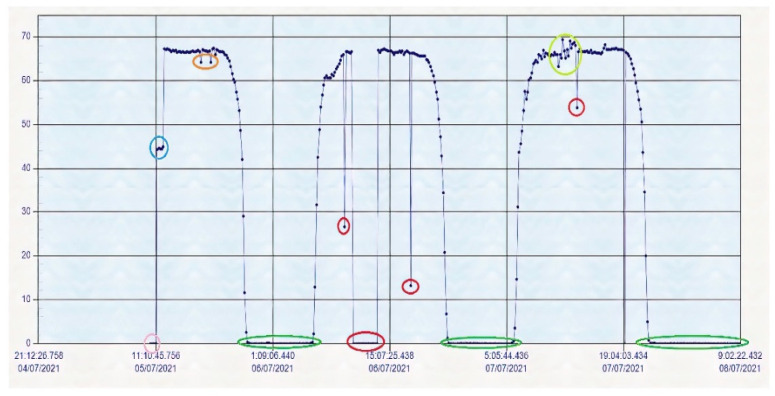
Historical data of the Voc solar panel string.

The SCADA also allows seeing the historical data Voc of the string of solar panels. A history record of less than 72 consecutive hours is shown in Figure 26.

Figure 26 shows multiple variations in the historical data of the voltage produced in open circuit by the string of the solar panels. The variations are indicated with ovals of different colours:

- pink when the solar panels are being connected;
- light blue when only two solar panels have been connected;
- orange when there are some small peaks that indicate that there are sporadic shadows from clouds;
- dark green indicates the night production, in this case 0 V;
- red indicates the loss of connection to the network;
- light green indicates the variation in solar irradiance.

A simple, easy to implement, reliable and efficient solution for predictive fault diagnosis in a string of solar panels has been proposed. This method has a very low implementation cost. Table 8 lists the materials and prices for implementing this methodology.

## 5. Conclusions

The main contribution of this work is that the diagnosis method is predictive, based on online detection by a predictor symptom parameter, sampled sequentially. It is centred in each solar panel of the PV string instead of being centred on the inverter. In this way, it makes easy immediately solar panel isolation. It is low-cost implementable, and integrable in actually working photovoltaic installations.

The application of predictive fault diagnosis in photovoltaic installations is an important factor in increasing the upward trend in the installation of this type of renewable energy. Achieving adequate levels of economic viability of this type of facility depends on obtaining an optimal production of electrical energy and the application of advanced predictive fault diagnosis techniques, which allow low-cost, immediate maintenance focused on each of the panels selectively.

The predictive fault diagnosis developed methodology, based on the use of the Voc-Isc parameters and focused on the solar modules, makes it possible that any symptom of power reduction, especially any shading process experienced on any panel of the solar installation, is detected and isolated immediately. Therefore, that reduction used comparatively can be employed as a predictor symptom for the early detection and isolation of the degrading event in the solar panel, and alternatively activate, by disconnecting it, the automatic interruption of the degrading accumulating effect on the affected panel, avoiding the appearance of an unrecoverable hot spot.

However, since the reduction in power is a symptom of other types of important failures that affect solar panels, the proposed methodology opens the possibility of carrying out in the best possible conditions another type of more specific redundant analysis based on visual and thermographic inspection, whose immediacy is not comparable to online Voc-Isc parametric diagnosis.

For the implementation of the predictive fault diagnosis algorithm, a specific architecture has been developed using low-cost sensors and devices to carry out online supervision of the fault predictor parameters by applying a sequentially sampling on each of the solar panels of the photovoltaic system strings. This architecture is integrable and scalable with real-world distributed control systems and conventional SCADAs for medium and large photovoltaic installations, using standard communication protocols that are easy to configure. It allows the use of different types of technologies, with low-cost implementation, integrable with purely industrial software that previously could only be connected by means of a PLC.

## Figures and Tables

**Table 1:** SPM04041200 solar panel features.

Parameter	Symbol	Value	Unit
Rated Maximum Power	Pmax	40	W
Tolerance	Tol	0 ± 3	%
Voltage at Pmax	Vmp	18.33	V
Current at Pmax	Imp	2.19	A
Open-Circuit Voltage	Voc	22.45	V
Short-Circuit Current	Isc	2.40	A
Nominal Operating Cell Temperature	NOCT	47 ± 2	°C
Maximum System Voltage		1000	V_DC_
Maximum Series Fuse Rating		10	A
Weight		3.10	Kg
Dimensions	425 × 668 × 25		mm
Operating Temperature	−40 to +85		°C
Application Class	Class A		
Protection Class	□		
Cell Technology	Mono-Si		

**Table 2:** Multiplexer/demultiplexer truth table.

S0	S1	S2	S3	ADC	Input Channel
# PIN	# PIN	# PIN	# PIN	1	None
0	0	0	0	0	0
1	0	0	0	0	1
0	1	0	0	0	2
1	1	0	0	0	3
0	0	1	0	0	4
1	0	1	0	0	5
0	1	1	0	0	6
1	1	1	0	0	7
0	0	0	1	0	8
1	0	0	1	0	9
0	1	0	1	0	10
1	1	0	1	0	11
0	0	1	1	0	12
1	0	1	1	0	13
0	1	1	1	0	14
1	1	1	1	0	15

**Table 3:** Operating ranges and sensitivity.

Part Number	T_A_ (°C)	Oprimized Range, Ip (A)	Sensitivity, Sens (Typ) (mV/A)
ACS712ELCTR-05A-T	−40 to 85	±5	185
ACS712ELCTR-20A-T	−40 to 85	±20	100
ACS712ELCTR-30A-T	−40 to 85	±30	66

**Table 4:** Software programs used.

Name	Version	Characteristics	Company
Arduino IDE	1.8.15	Open source	Arduino
		Easy to program	
		Versatile for programming other modules.	
KEPServerEX	6.4.321.0	Versatility to unite various communication technologies.	Kepware
		Security in communications.	
		It brings together several industrial technologies.	
		Secure communication with software for the development of SCADA systems.	
iFIX	6.5.	HMI/ SCADA development.	General Electric
		High performance in Monitoring and Control.	
		Greater efficiency in operations.	

**Table 5:** Voc measurements in solar panels.

SP	Multimeter (V)	Sensor (V)	Error	%
String SP	66.6	67.08	0.48	0.72%
SP 1	22.1	22.05	0.05	0.23%
SP 2	22.10	22.10	0.00	0.00%
SP 3	22.2	22.15	0.05	0.23%

**Table 6:** Isc measurements in solar panels.

SP	Multimeter (A)	Sensor (A)	Error	%
SP 1	2.14	2.13	0.01	0.47%
SP 2	2.23	2.25	0.02	0.90%
SP 3	2.24	2.23	0.01	0.45%

**Table 7:** Shaded Areas.

	L1 (cm)	L2 (cm)	Area (m^2^)	%
Solar panel	37	63.5	0.235	100.00%
Shadow 1	4.7	4	0.002	0.80%
Shadow 2	16	3.7	0.006	2.52%
Shadow 3	37	3.7	0.014	5.98%
Shadow 4	63	4	0.025	10.73%
Shadow 5	20.5	16.3	0.033	14.22%
Shadow 6	37	16.5	0.061	25.98%
Shadow 7	46	33.2	0.153	65.00%

**Table 8:** Material costs to implement the proposed solution.

Description	Units	Unit Price (€)	Total Price (€)
ESP8266 Module	1	2.31	2.31
CD74HC4067 Module	1	0.44	0.44
ACS712ELCTR-05B-T Sensor	1	0.83	0.83
FZ0430 Sensor	2	0.21	0.42
Relay Module	2	3.55	7.10
Resistor 22 kΩ	1	0.05	0.05
Metal film resistor 133 kΩ	1	0.10	0.10
Male and female waterproof connector	3	0.16	0.48
Terminal blocks	4	0.17	0.68
Breadboard	1	0.96	0.96
Perforated breadboard	1	0.58	0.58
**Total**			**13.95**

## Data Availability

Not applicable.

## Abbreviations

ADC	Analog-to-Digital Converter
GPIOS	General purpose input/output interface
I	Current
Isc	Short circuit current
IoT	Internet of things
I2C	Interface inter-integrated circuit
I2S	Interface Integrated Interchip Sound
MQTT	Message Queue Telemetry Transport
OPC-OLE	Object Linking and Embedding for process control
OPC-UA	Unified Architecture OPC
PLC	Programmable Logic Controller
PVM	Photovoltaic module
PWM	Pulse-Width Modulation
SDIO	Safe digital input/output interface
SP	Solar panel
SPI/HSPI	Serial Peripheral Interface
UART	Universal Asynchronous Receiver Transmitter
V	Voltage
V_DC_	Direct Current Voltage
Voc	Open circuit voltage
wsd	Wind speed and direction
